# Supplementary figures and images for: TruD technology for the study of epi- and endothelial tubes in vitro
Source: PLoS One. 2024 May 10;19(5):e0301099. doi: 10.1371/journal.pone.0301099 (PMC11086873; doi:10.1371/journal.pone.0301099)

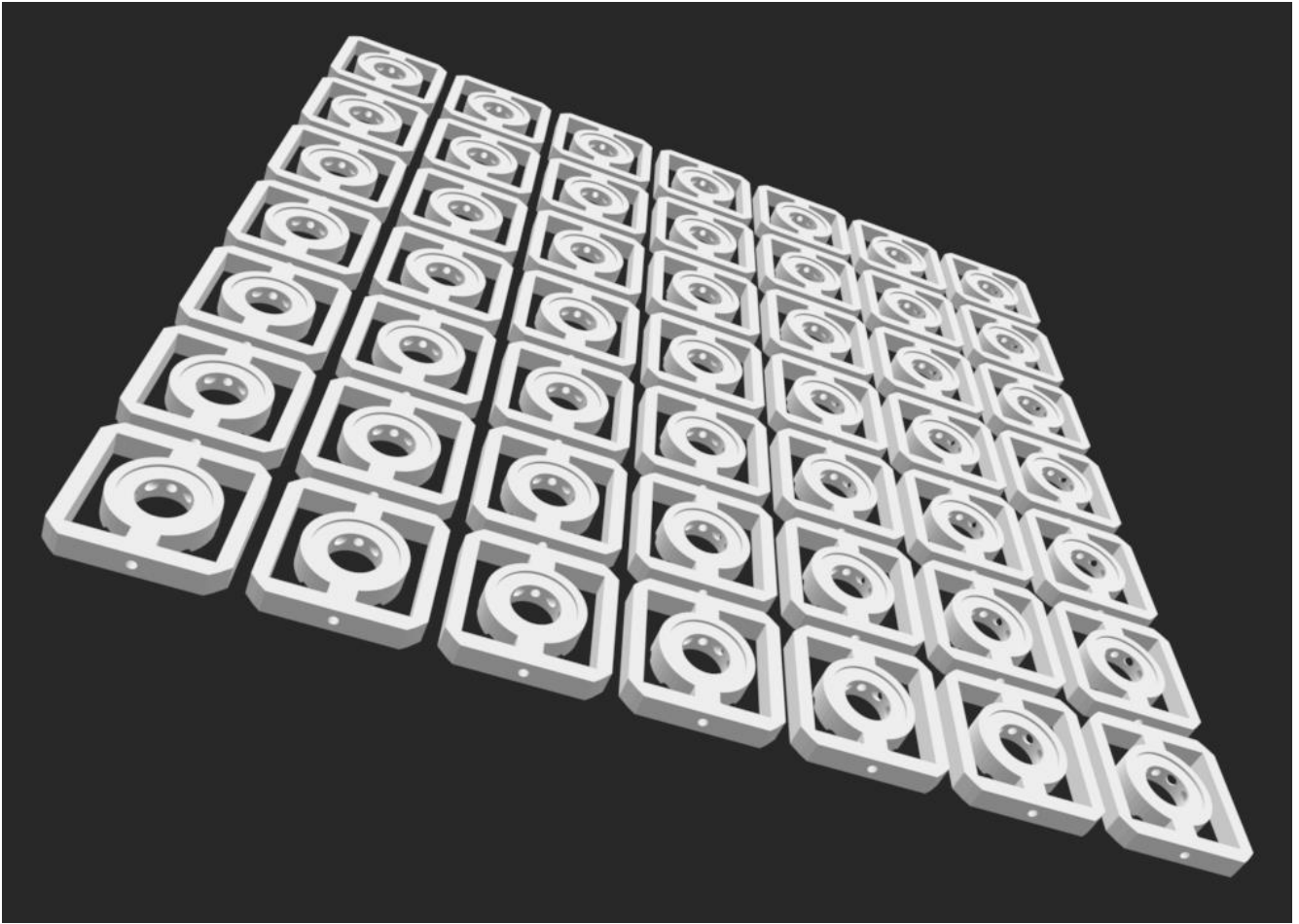

**S1 Fig. Basic TruD chip.**

Supplement: S1 Fig — (PDF) [file pone.0301099.s001.pdf]

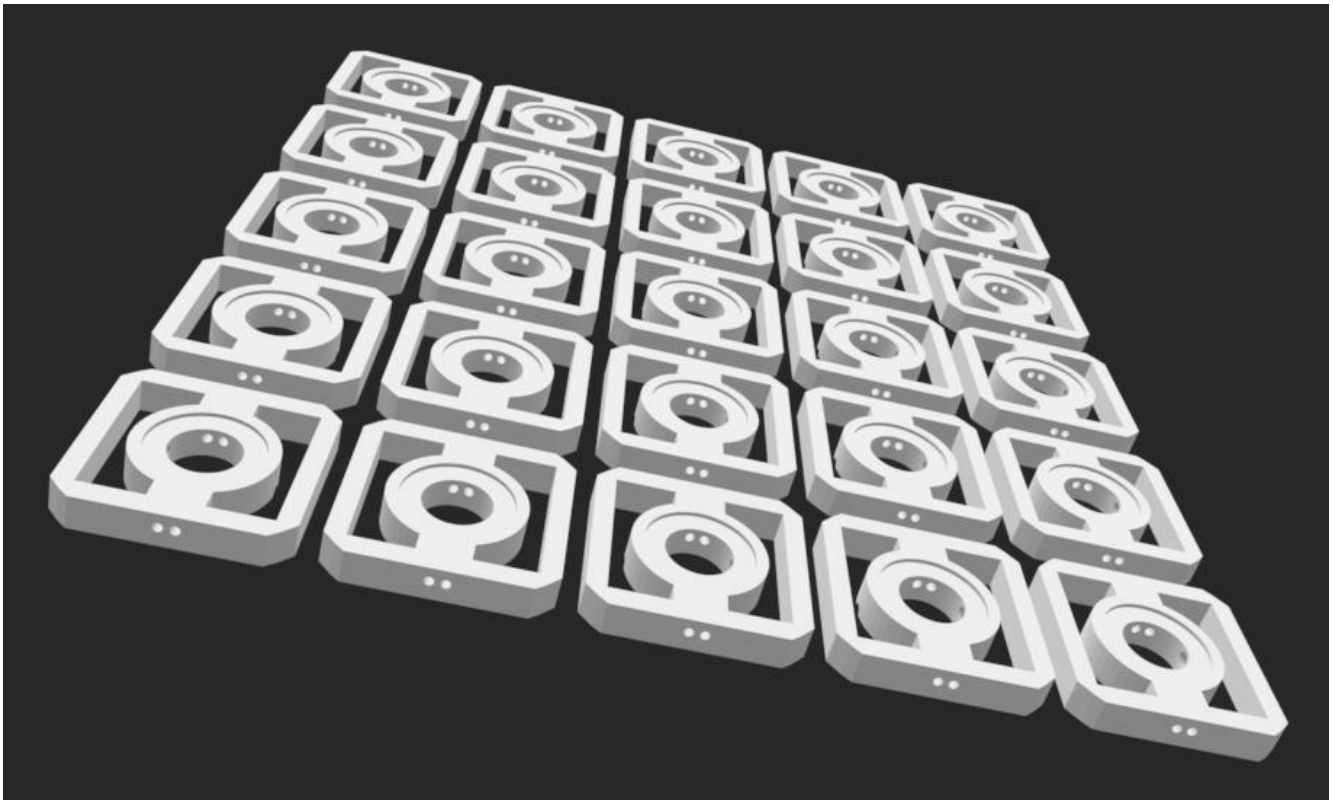

**S2 Fig. TruD chip with parallel pores.**

Supplement: S2 Fig — (PDF) [file pone.0301099.s002.pdf]

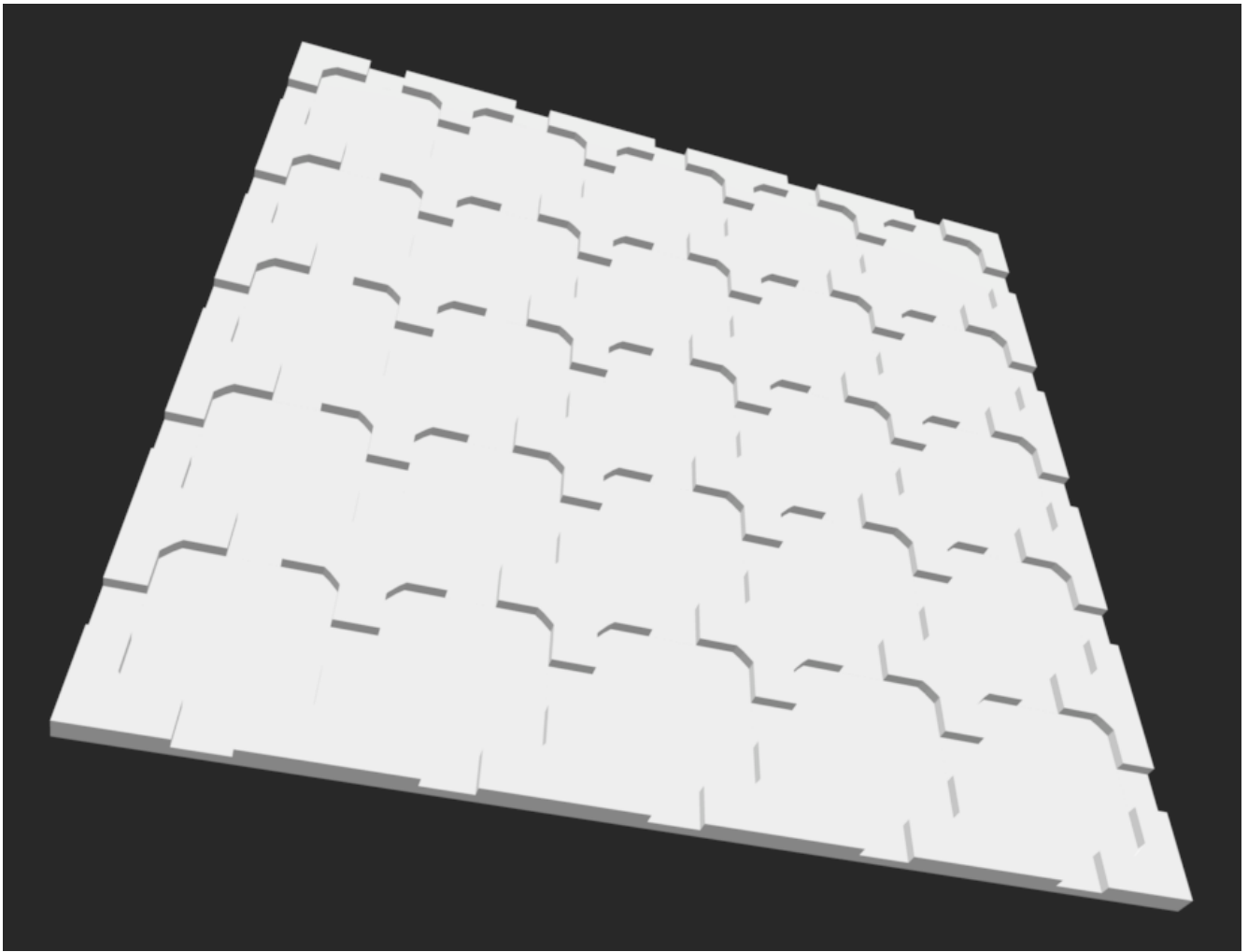

**S6 Fig.** Tray for handling chips while mounting coverslips and for storage prior to usage.

Supplement: S6 Fig — (PDF) [file pone.0301099.s006.pdf]

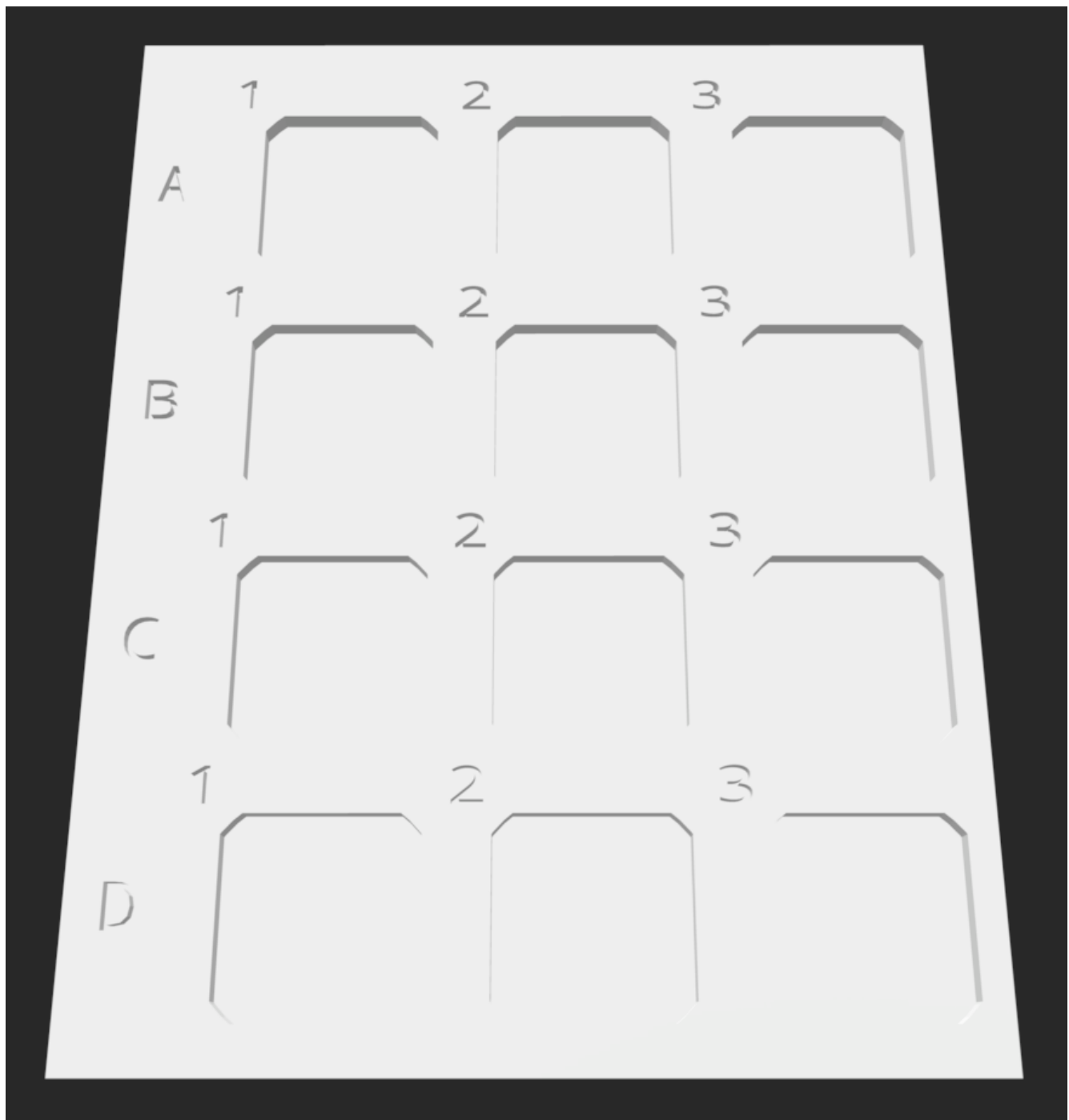

**S8 Fig. Alphanumeric holder for enabling easy transport and short-term storage of TruD chips.**

Supplement: S8 Fig — (PDF) [file pone.0301099.s008.pdf]

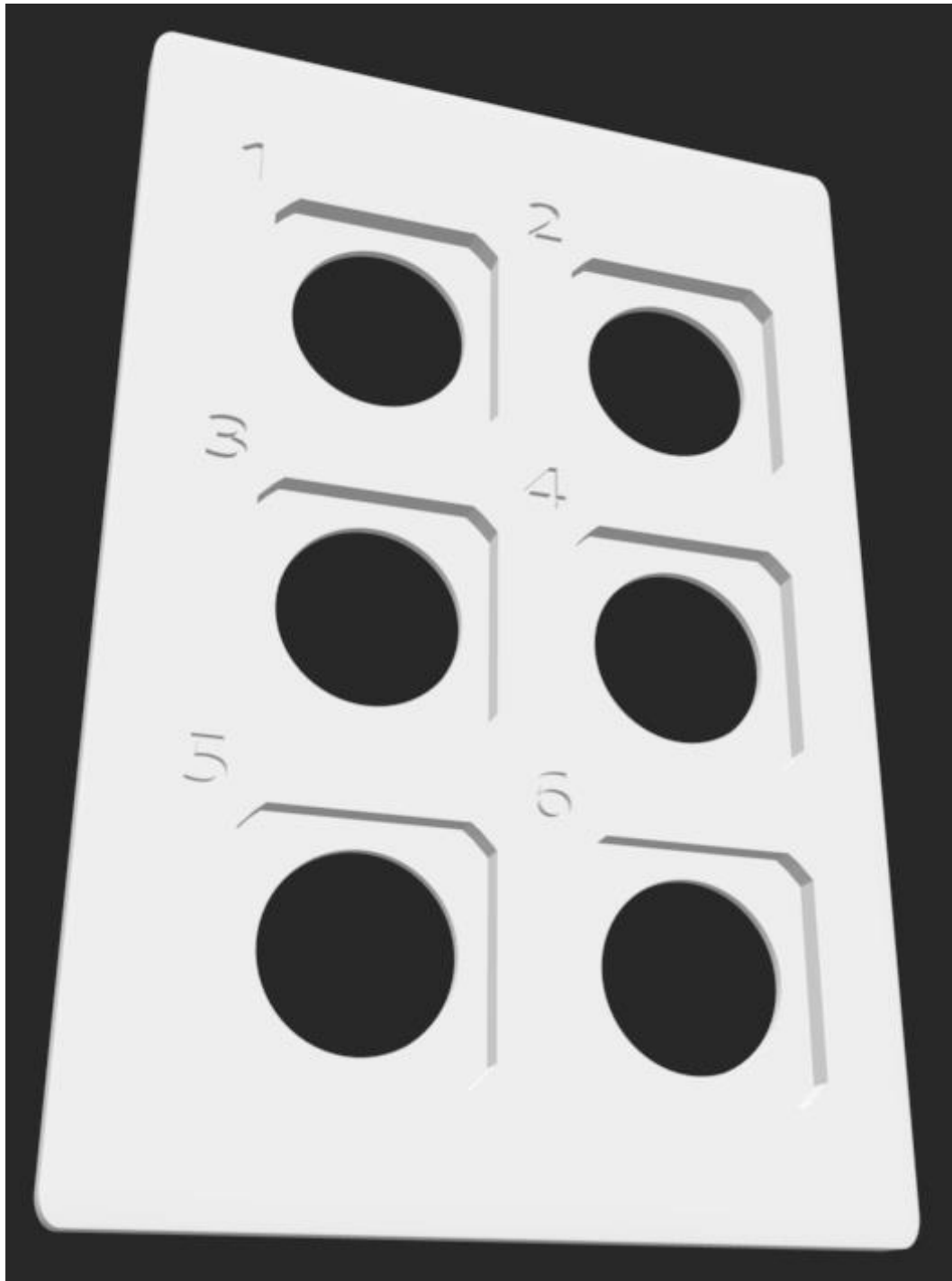

**S9 Fig. TruD adaptor for Keyence microscope.**

Supplement: S9 Fig — (PDF) [file pone.0301099.s009.pdf]

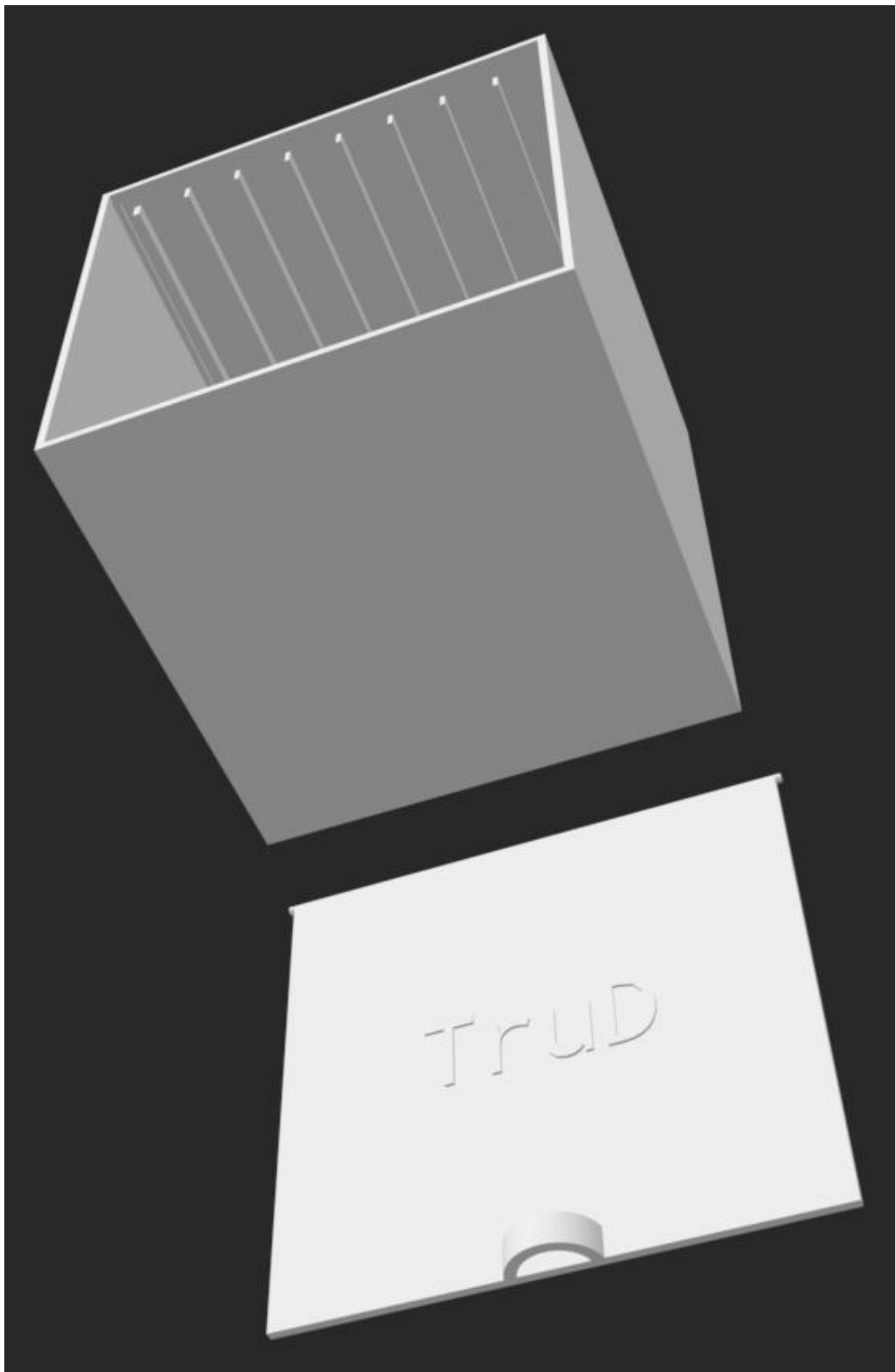

**S10 Fig. Cabinet for dust-free storage of TruD chips.**

Supplement: S10 Fig — (PDF) [file pone.0301099.s010.pdf]

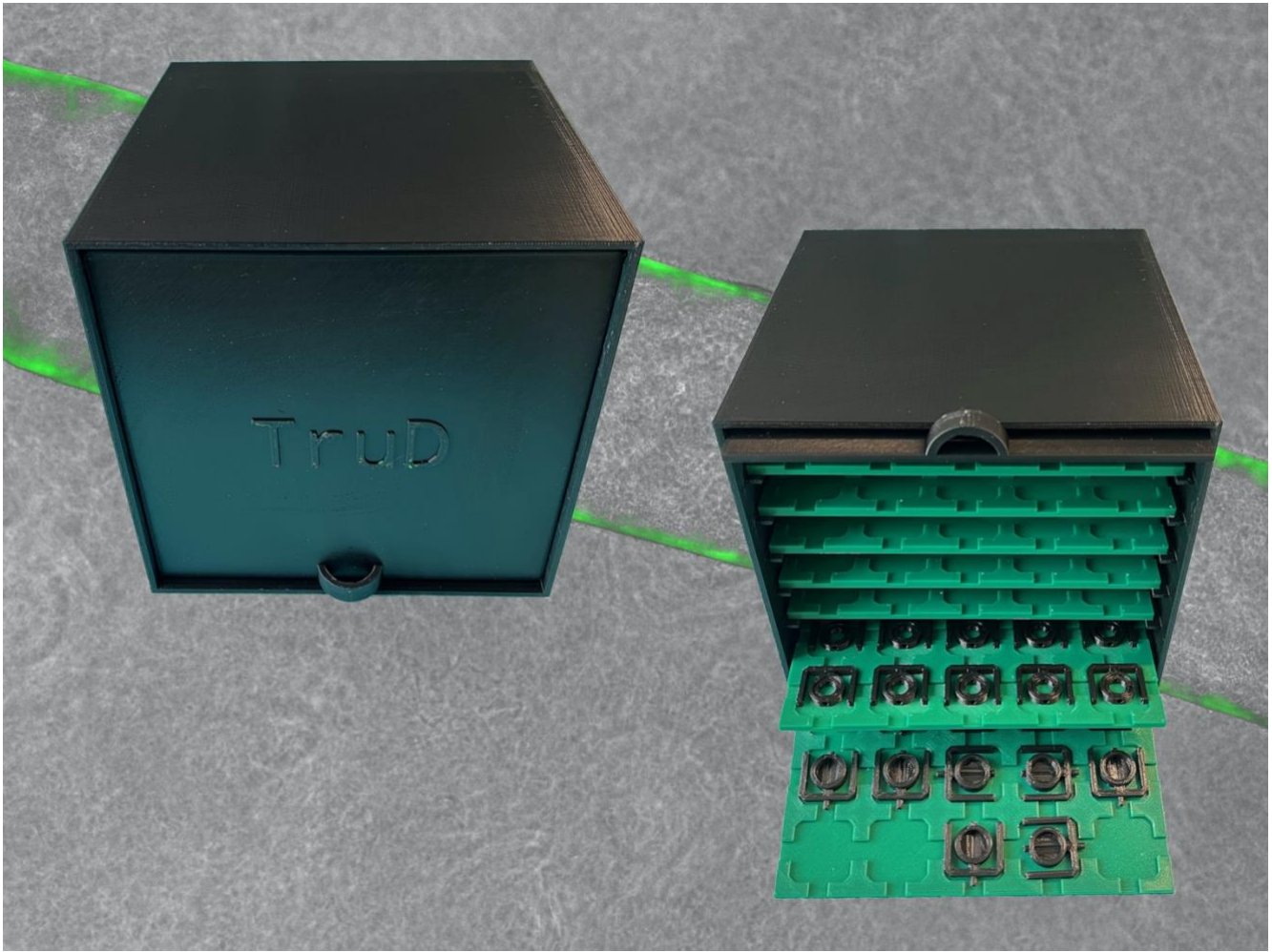

**S12 Fig. 3D print of the cabinet 10.stl file shown in S10 Fig.**

Supplement: S12 Fig — (PDF) [file pone.0301099.s012.pdf]
