# Supplementary material for: TruD technology for the study of epi- and endothelial tubes in vitro
Source: PLoS One. 2024 May 10;19(5):e0301099. doi: 10.1371/journal.pone.0301099 (PMC11086873; doi:10.1371/journal.pone.0301099)
Supplement: S3 Fig — Note, the print support below the tubular extension is removed prior to usage. (PDF) [file pone.0301099.s003.pdf]

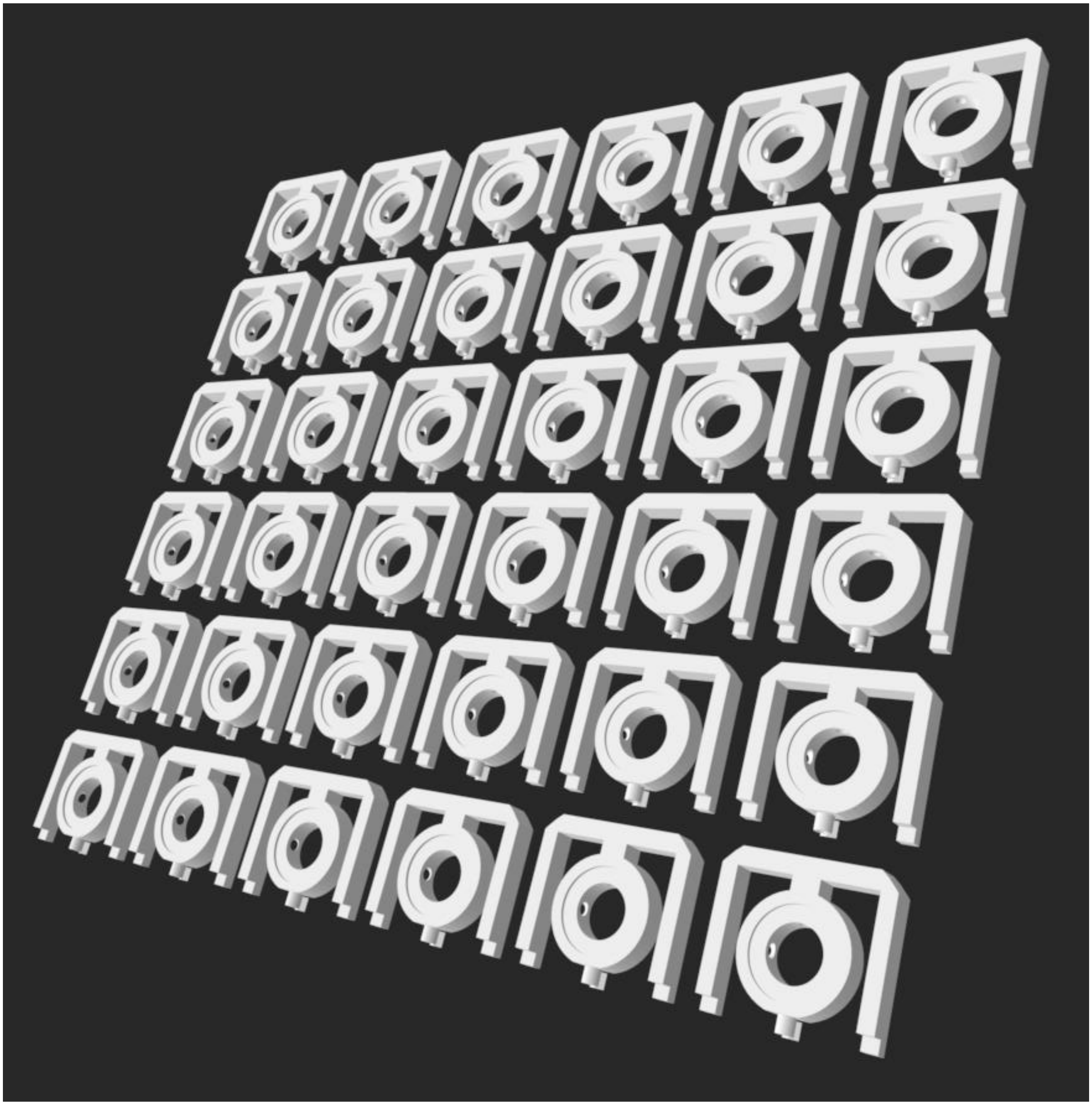

S3 Fig. Male TruD chip. Note, the print support below the tubular extension is removed prior to usage.
