# Supplementary material for: TruD technology for the study of epi- and endothelial tubes in vitro
Source: PLoS One. 2024 May 10;19(5):e0301099. doi: 10.1371/journal.pone.0301099 (PMC11086873; doi:10.1371/journal.pone.0301099)
Supplement: S4 Fig — Note, the print supports beneath the external walls are removed prior to usage. (PDF) [file pone.0301099.s004.pdf]

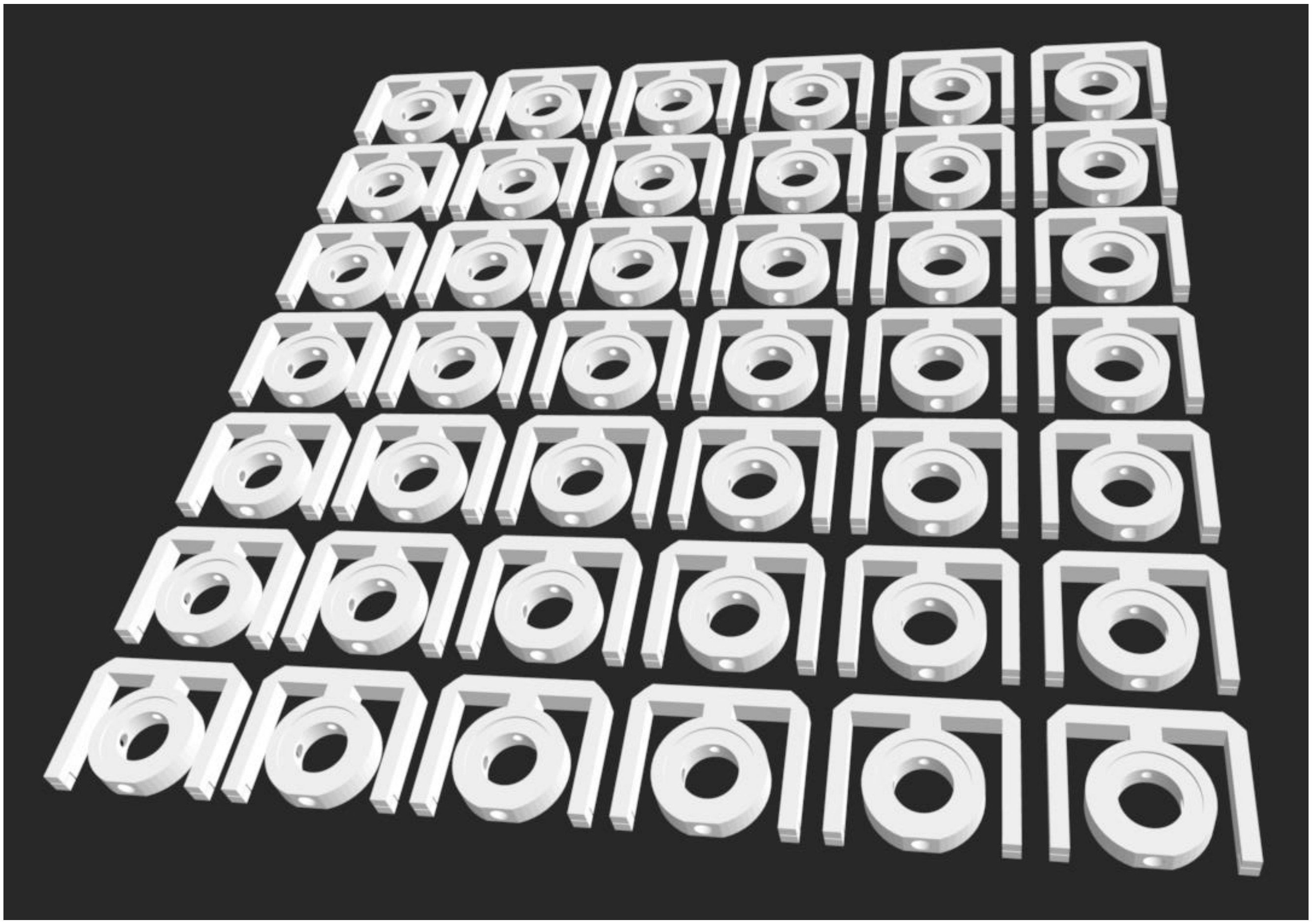

S4 Fig. Female TruD chip. Note, the print supports beneath the external walls are removed prior to usage.
