# Supplementary material for: TruD technology for the study of epi- and endothelial tubes in vitro
Source: PLoS One. 2024 May 10;19(5):e0301099. doi: 10.1371/journal.pone.0301099 (PMC11086873; doi:10.1371/journal.pone.0301099)
Supplement: S5 Fig — Print supports, as described in legends to S3 and S4 Figs, are removed prior to usage. (PDF) [file pone.0301099.s005.pdf]

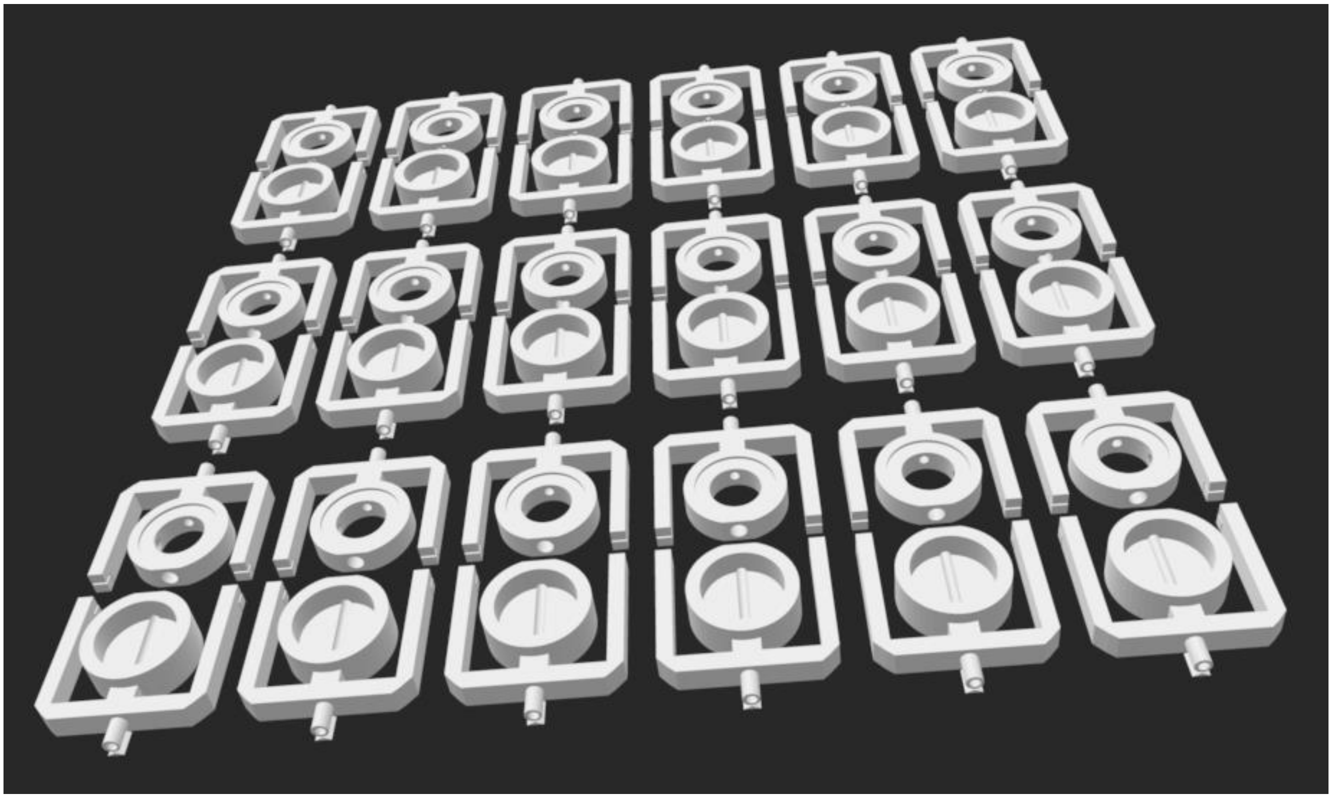

**S5 Fig. Female chip and male TruD chips of which the latter is modified with a reservoir to permit flow.** Print supports, as described in legends to S3 and S4 Figs, are removed prior to usage.
