# Supplementary material for: TruD technology for the study of epi- and endothelial tubes in vitro
Source: PLoS One. 2024 May 10;19(5):e0301099. doi: 10.1371/journal.pone.0301099 (PMC11086873; doi:10.1371/journal.pone.0301099)
Supplement: S7 Fig — The dimensions of the rack match the bottom of a 6-well dish such that the lids can be used to cover the chips to avoid contamination while the ECM solidifies after placement in the incubator. (PDF) [file pone.0301099.s007.pdf]

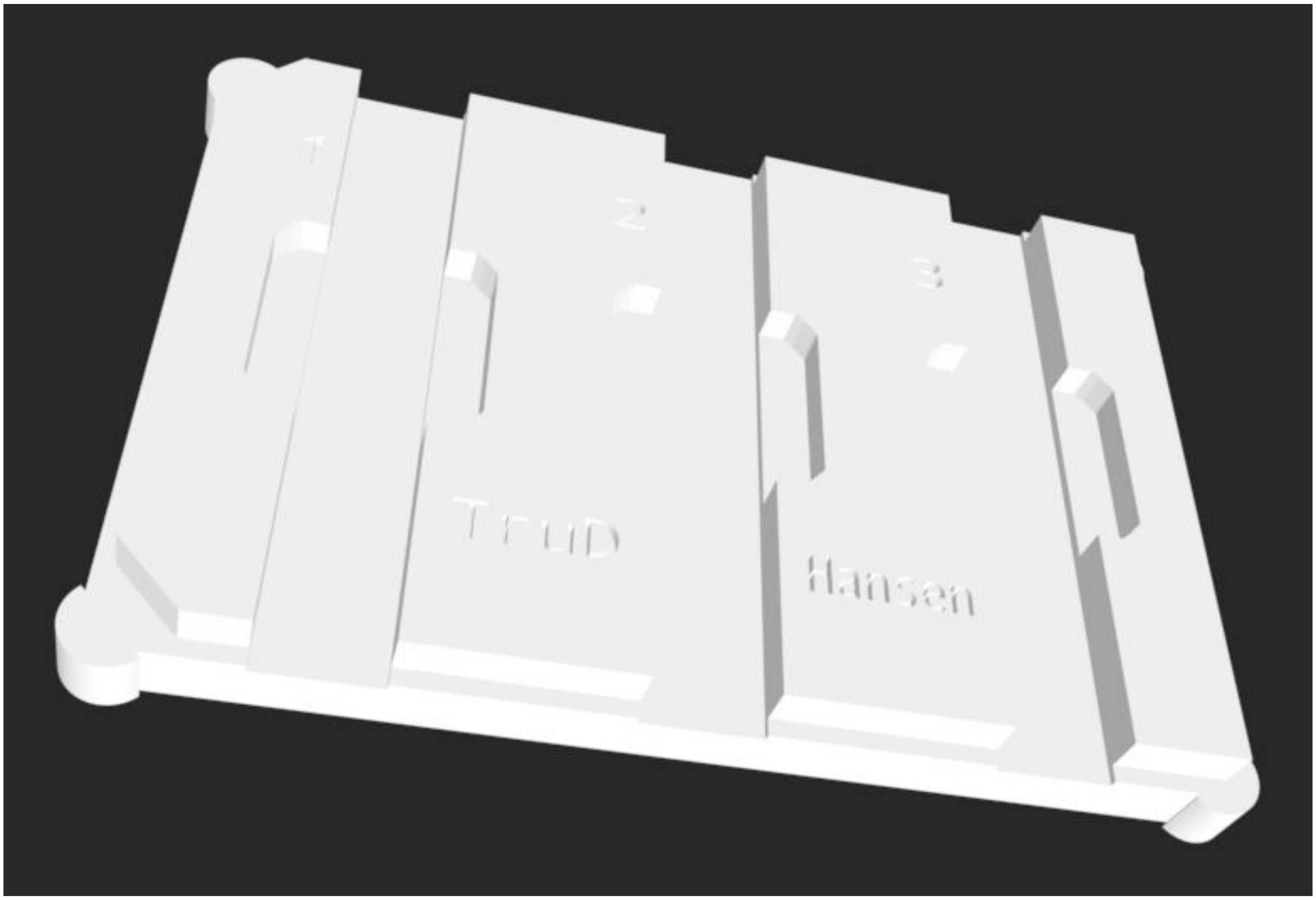

S7 Fig. Rack for facilitating handling of TruD chips during pipetting of ECM as well as injection of cells. The dimensions of the rack match the bottom of a 6-well dish such that the lids can be used to cover the chips to avoid contamination while the ECM solidifies after placement in the incubator.
