# Supplementary material for: TruD technology for the study of epi- and endothelial tubes in vitro
Source: PLoS One. 2024 May 10;19(5):e0301099. doi: 10.1371/journal.pone.0301099 (PMC11086873; doi:10.1371/journal.pone.0301099)
Supplement: S11 Fig — (A) Tray for mounting coverslips and storage. (B) Casting rack. (C) Alphanumeric holder. (D) Adaptor for Keyence microscope. (PDF) [file pone.0301099.s011.pdf]

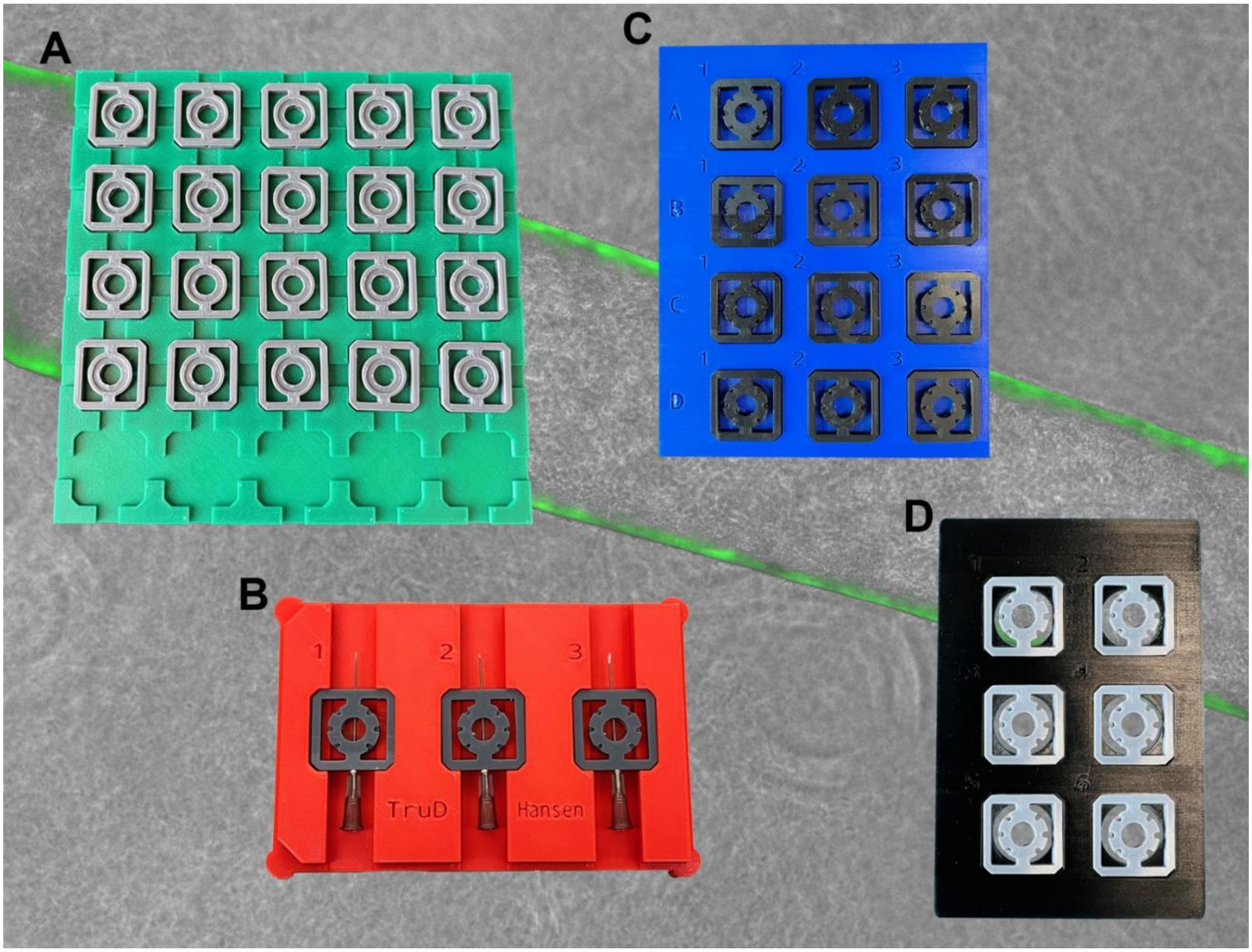

S11 Fig. Examples of 3D prints of the .stl files illustrated in S6-9 Figs. (**A**) Tray for mounting coverslips and storage. (**B**) Casting rack. (**C**) Alphanumeric holder. (**D**) Adaptor for Keyence microscope.
